# Creative Arts Interventions in Addressing Depression in Older Adults: A Systematic Review

**DOI:** 10.1093/geroni/igab046.2171

**Published:** 2021-12-17

**Authors:** Nisha Sajnani, Kim Dunphy, Felicity Baker, Ella Dumaresq, Katrina Caroll-Haskins, Jasmin Eickholt, Maya Ercole, Girija Kaimal

**Affiliations:** 1 New York University, New York City, New York, United States; 2 University of melbourne, University of Melbourne, Victoria, Australia; 3 Holy Family University, Philadelphia, Pennsylvania, United States; 4 University of Applied Sciences, Würzburg, Bayern, Germany; 5 Drexel University, Philadelphia, Pennsylvania, United States

## Abstract

Depression experienced by older adults is proving an increasing global health burden, with rates as high as 27% in the USA. This is likely to increase in coming years as the number and proportion of older adults in the global population rises. Therefore, it is imperative that the effectiveness of approaches to the prevention and treatment of depression are understood. Creative arts interventions, including art, dance movement, drama, and music, are utilized internationally to reduce depressive symptoms in older adults and promote wellbeing. This includes interventions led by trained arts therapists as well as other health and arts professionals. This presentation will include a report of findings from a recent systematic review of the outcomes of four creative arts modalities (art, dance movement, drama, and music) with particular attention paid to processes of change documented in each modality.

